# Model based approach for estimating the dosage regimen of indomethacin a potential antiviral treatment of patients infected with SARS CoV-2

**DOI:** 10.1007/s10928-020-09690-4

**Published:** 2020-05-20

**Authors:** Roberto Gomeni, Tianhong Xu, Xuejuan Gao, Françoise Bressolle-Gomeni

**Affiliations:** 1 Pharmacometrica, Lieu-Dit Longcol, 12270 La Fouillade, France; 2 BaylorOracle Inc., Hangzhou, China

**Keywords:** SARS-CoV-2, Indomethacin, Model-based, Clinical dose, Translational model

## Abstract

To face SARS-CoV-2 pandemic various attempts are made to identify potential effective treatments by repurposing available drugs. Among them, indomethacin, an anti-inflammatory drug, was shown to have potent in-vitro antiviral properties on human SARS-CoV-1, canine CCoV, and more recently on human SARS-CoV-2 at low micromolar range. Our objective was to show that indomethacin could be considered as a promising candidate for the treatment of SARS-CoV-2 and to provide criteria for comparing benefits of alternative dosage regimens using a model-based approach. A multi-stage model-based approach was developed to characterize % of recovery and viral load in CCoV-infected dogs, to estimate the PK of indomethacin in dog and human using published data after administration of immediate (IR) and sustained-release (SR) formulations, and to estimate the expected antiviral activity as a function of different assumptions on the effective exposure in human. Different dosage regimens were evaluated for IR formulation (25 mg and 50 mg three-times-a-day, and 25 mg four-times-a-day), and SR formulation (75 mg once and twice-a-day). The best performing dosing regimens were: 50 mg three-times-a-day for the IR formulation, and 75 mg twice-a-day for the SR formulation. The treatment with the SR formulation at the dose of 75 mg twice-a-day is expected to achieve a complete response in three days for the treatment in patients infected by the SARS-CoV-2 coronavirus. These results suggest that indomethacin could be considered as a promising candidate for the treatment of SARS-CoV-2 whose potential therapeutic effect need to be further assessed in a prospective clinical trial.

**Electronic supplementary material:**

The online version of this article (10.1007/s10928-020-09690-4) contains supplementary material, which is available to authorized users.

## Introduction

To face SARS-CoV-2 pandemic various attempts are made to identify potential effective treatments by repurposing available drugs. Among these treatments, indomethacin (INDO), a potent anti-inflammatory and antipyretic drug acting on COX1/2 enzymes, developed for the treatment of rheumatoid arthritis in the 1960s [1, 2] seems to have the potential to become an effective treatment for the patients infected with SARS CoV-2. INDO can be used not only for its antipyretic properties, common to other non-steroidal analgesic drugs, as symptomatic treatment [3, 4], but also to improve oxygenation in patients with acute respiratory distress syndrome [5, 6] and to robustly reduce proinflammatory interleukin-6 levels [7, 8].

Following single oral immediate-release (IR) doses of 25 mg or 50 mg, INDO is readily absorbed, attaining peak plasma concentrations at about 2 h post-dose. Orally administered INDO IR is almost completely bioavailable, with 90% of the dose absorbed within 4 h. INDO sustained-release (SR) 75 mg is designed to release 25 mg of the drug initially and the remaining 50 mg over approximately 12 h (90% of dose absorbed by 12 h). INDO is highly bound to protein in plasma (about 99%) over the expected range of therapeutic plasma concentrations and it has been found to cross the blood–brain barrier and the placenta, and appears in breast milk. INDO exists in plasma as parent drug and its desmethyl, desbenzoyl, and desmethyldesbenzoyl metabolites, all in the unconjugated form. INDO is eliminated via renal excretion, metabolism, and biliary excretion with a mean half-life of about 4.5 h.

The following dosage regimens are recommended in the FDA label for the different indications of INDO IR [9]: INDO 25 mg twice a day or three times a day. If this is well tolerated, increase the daily dosage by 25 mg or by 50 mg, until a total daily dose of 150–200 mg in 3 or 4 divided doses is reached.

INDO SR [10], 75 mg once a day can be substituted for INDO IR capsules, 25 mg three times a day. However, there will be significant differences between the two dosage regimens in INDO blood levels, especially after 12 h. In addition, INDO SR, 75 mg twice a day can be substituted for INDO IR capsules, 50 mg three times a day.

In 2006, INDO has been shown to have antiviral properties against coronaviruses, including human SARS-CoV-1 and canine coronavirus (CCoV) [11]. The study, conducted in dogs, identified INDO as a potent inhibitor of coronavirus replication and suggested that, having both anti-inflammatory and antiviral activities, INDO could be beneficial in SARS therapy. In this study, the in-vivo antiviral efficacy was determined by evaluating virus titres in CCoV-infected dogs treated orally with INDO 1 mg/kg body weight, the therapeutic dose in human [12] and dog [13]. This dosage regimen was associated with a dramatic decrease in virus titres in the feces of the treated animals for 4 days.

The antiviral activity of INDO was shown to be associated with the activation of the double‐stranded RNA (dsRNA)‐dependent protein kinase R (PKR). INDO, at concentrations in the low micromolar range, activates PKR leading to an interferon‐ and dsRNA‐independent phosphorylation of the eukaryotic initiation factor‐2 α‐subunit [14], shutting off viral protein translation and blocking viral replication [15]. A recent study conducted in-vitro and in CCoV infected dogs showed that INDO has also a direct and potent antiviral activity against the SARS CoV-2 pseudovirus [16]. In this study the rate of recovery in CCoV-infected dogs treated orally with INDO 1 mg/kg body weight was also evaluated. These new data indicated that INDO has a potency comparable to the potency of hydroxychloroquine (HCQ) [17] and remdesivir [18] and that INDO could be a very effective potential treatment of patients affected by the SARS CoV-2.

It is important to note that in April 2020 an open-label, single-arm, phase II study to evaluate the efficacy and safety of oral HCQ, INDO and azithromycin in subjects positive with SARS-CoV-2 with mild symptoms was started. This study was posted in clinicaltrials.gov with reference NCT04344457 [19]. This study, expected to include 80 subjects, will assess the improvement of SARS-CoV-2 disease status as measured by time (days) required to improve the clinical status from mild to symptom-free using oral HCQ 200 mg twice a day (BID) for 5 days; oral indomethacin 50 mg three times a day (TID) for 14 days; and oral azithromycin 500 mg once a day (QD) for 3 days. Overall, these data suggest that INDO could exert both anti-inflammatory and antiviral effects for the treatment of patients infected with SARS-CoV-2.

The objective of the present paper was to apply a meta-analytic model-based approach using published data to estimate the relationship between INDO exposure and response in dogs after administration of an IR formulation and to extrapolate this relationship to humans after IR and SR formulations. The exposure–response model will be used for predicting the most effective dosage regimen of INDO suitable for maximizing the potential clinical benefit for the treatment of patients infected by the SARS-CoV-2 coronavirus.

## Methods

### Anti-coronavirus activity in-vitro

INDO was shown to inhibit SARS CoV-1 virus replication with an inhibitory concentration 50% (IC50) of about 5 µM (i.e. 1.79 mg/L), while aspirin was ineffective [11]. In a more recent study, INDO was shown to inhibit SARS CoV-2 pseudovirus replication [16]. In this recent study, the pseudovirus model that contains the SARS-CoV-2 spike was used (SARS CoV-2 GenBank: MN908947.3). Briefly, African green monkey kidney VERO E6 cells were infected with SARS CoV-2 pseudovirus and treated with different concentrations of INDO (0, 0.1, 1, 5, 10, 50, 100, 500 μM) or aspirin (0, 0.1, 1, 5, 10, 50, 100, 500 μM) as control at 48 h post infection. The level of cell infection was determined by luciferase activity in the cell lysates of infected cells at 48 h post-infection (p.i.). INDO was found to possess a remarkable antiviral activity, reducing viral particle production dose-dependently with an IC50 of ~ 1 μM (i.e. 0.358 mg/L), and selective index of 500, and caused a dramatic reduction relative light unit to zero at 48 h p.i, in VERO E6 cells [16].

### Anti-coronavirus activity in CCoV-infected dog

Two different studies were conducted to evaluate whether INDO could be effective in vivo. In the first study [11], the in-vivo antiviral efficacy of INDO was assessed by evaluating the CCoV viral load in infected dogs treated with INDO. Dogs were tested for the presence of CCoV RNA in feces by a real-time RT-PCR assay and CCoV antibodies in serum samples by an ELISA test. Dogs were treated orally with INDO (1 mg/kg body weight) daily for 4 days, starting on day 4 p.i., whereas dogs in a separate group served as infected non-treated controls. In INDO-treated dogs, viral RNA titres in the feces decreased rapidly after starting treatment, reaching minimal levels at day 7 p.i. in concomitance with the peak observed in non-treated dogs. INDO antiviral effect was reversed upon suspension of treatment demonstrating a potent anti-coronavirus activity of INDO in vivo.

In the second study [16], the % of recovery in CCoV-infected dogs treated orally with INDO 1 mg/kg body weight was evaluated. Dogs treated with ribavirin (a broad-spectrum antiviral drug with efficacy against RNA and DNA viruses) were used as a control group. The enrolled dogs were confirmed for diagnosis of CCoV infection with a canine coronavirus test kit and between 2–3 days of the onset of symptoms. The time of recovery was determined by the disappearance of symptoms and a negative diagnosis by the canine coronavirus test kit [20]. In INDO-treated dogs, a complete recovery was observed after 5 days of treatment.

### Modeling strategy

**Fig. 1:**
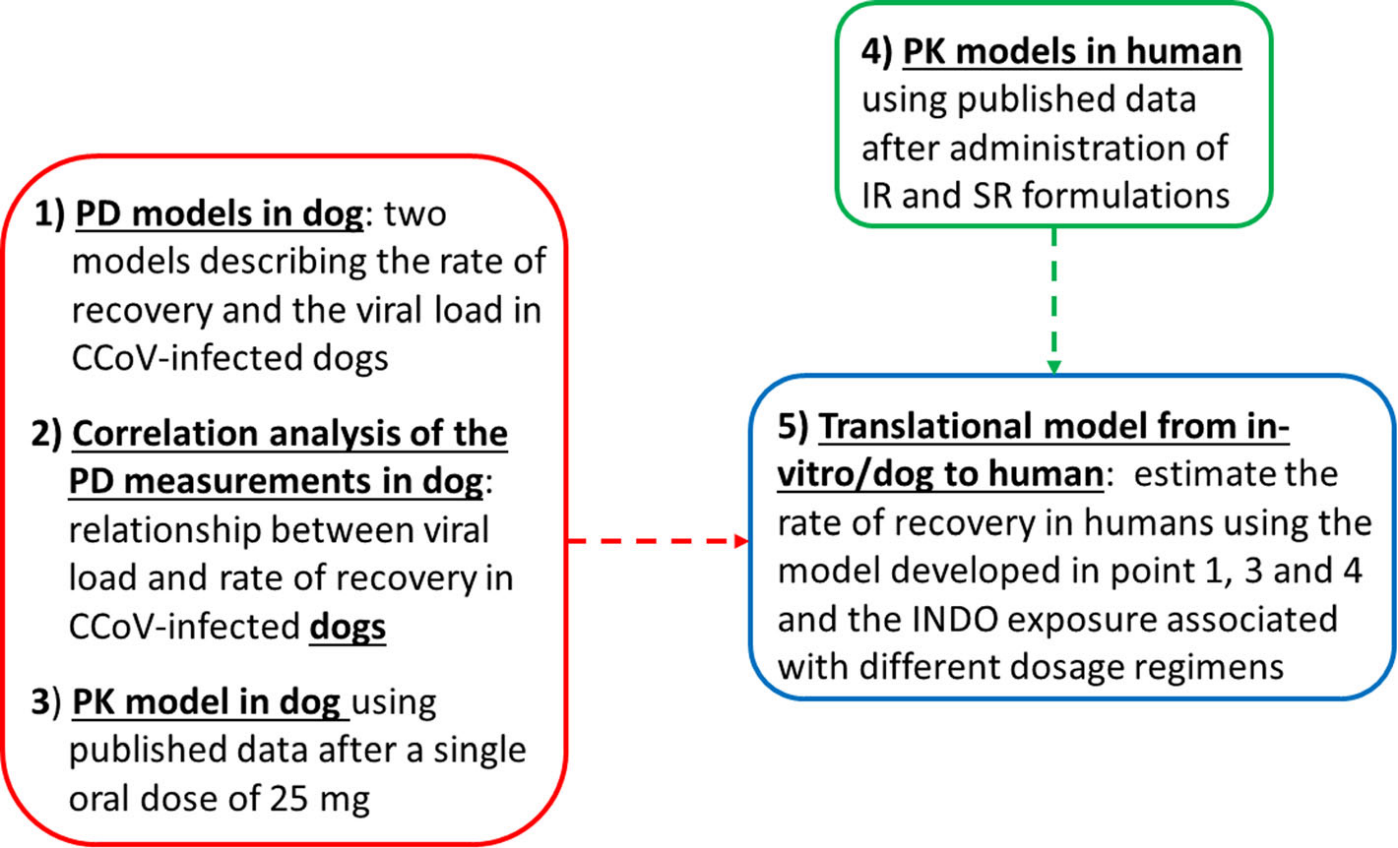
Multi-stage model-based approach

The modeling strategy was based on the characterization of the time course of response (% of viral load inhibition and % of recovery) in dogs and in the evaluation of the relationship between INDO exposure (derived from published data) and time course of the response. The objective of this analysis was to show that the viral load inhibition was the driver of the % recovery by combining the results of different studies. The % of recovery in dog was assumed to be driven by the time during which the exposure remained above an effective concentration value. Finally, the human PK following IR and SR formulations was derived from published data and a translational model was developed for estimating the clinical response in human based on the expected time during which the human exposure remained above the effective concentration following different dosage regimens. A multi-stage model-based approach was developed as illustrated in Fig. 1.

#### Modeling rate of recovery and viral load in dog

The rate of recovery and the viral load inhibition in CCoV-infected dogs were expressed in % ranging from 0 (% recovery at baseline or full viral inhibition) to 100% (full recovery or baseline viral load). The rate of recovery and the viral load inhibition were modeled as a function of time with the assumption that the effect was driven by the constant INDO exposure maintained for 5 days. The following Weibull models were used:

1 $${\text{Recovery}}\left( \% \right) = 100 \times \left( {1 - e^{{ - \left( {\frac{{{\text{time}}}}{{td}}} \right)g}} } \right)$$

2 $$\text{Viral load inhibition} \left(\%\right)=100\times {e}^{{-(\frac{\text{time}}{td})}^{g}}$$

where td is the time to response defined as the time necessary for recovery or for inhibition of 63% of the baseline values and g is the shape of the response.

#### PK in dog and human

INDO PK was evaluated in Beagle dogs weighing from 8 to 10 kg at a single dose of 25 mg. Blood samples were collected at 0.333, 0.667, 1, 1.5, 2–4, 6, 8, 10 and 12 h after administration of uncoated pellets [21].

Human INDO IR PK was evaluated at single doses of 25 mg, 50 mg, and 75 mg in 8 healthy volunteers, and 100 mg in 4 healthy volunteers and 4 patients with rheumatoid arthritis. Blood samples were taken at intervals for up to 7.5 h after drug intake. The results of the study indicated no major differences between PK in healthy subjects and patients [22].

The INDO SR at the dose of 75 mg is a formulation designed to IR 25 mg and to provide a delayed release of the remaining 50 mg of the dose. PK samples collected at intervals for up to 12 h in 14 subjects were used for the assessment of the PK characteristics of INDO SR [23].

The mean concentrations time course of INDO in dog and human were obtained by digitizing the concentration versus time graphs reported in the referred publications.

#### Translational model

INDO is a weak organic acid with a molecular weight of 357.8 g/mol that is 99% bound in dog and human to plasma albumin but not to red blood cells [24]. Data from in vitro plasma protein binding experiments are frequently used to guide the estimate of in vivo efficacy in the assumption that the efficacy is driven by the free (unbound) drug concentration [25]. As the protein binding in human and dog was the same (i.e. 99%), the free fraction was also the same. As a consequence, the translational model was based on total INDO concentrations.

Two simulation scenarios were considered assuming that the effective INDO concentration in human was driven by the in-vitro potency associated with: 1) 50% of the inhibition effect (IC50), and 2) 95% of the inhibition effect (IC95).

The estimated effective antiviral concentration in human is usually defined as the INDO concentration at which virus replication is inhibited by 50% (i.e. the IC50 value). However, a more aggressive and more clinically appropriate target such as IC90 was proposed for any repurposed drug against SARS-CoV-2 [26]. Therefore, as recommended for other antiviral treatments, two target inhibitory concentrations were retained: IC50 (corresponding to an exposure of 0.358 mg/L) and IC95 (corresponding to an exposure of 1.074 mg/L) [27].

The time during which INDO concentration remains above the effective concentration was used as a driver of the response, as commonly done for the assessment of the relationship between pharmacokinetics and pharmacodynamics of antimicrobial agents [28]. Three dosage regimens were evaluated for the IR formulation: 50 mg three-times-a-day, 25 mg three-times-a-day, and 25 mg four-times-a-day. Two dosage regimens were evaluated for the SR formulation: 75 mg once-a-day, and 75 mg twice-a-day.

The extrapolation of the relationship between in-vivo response in dog to human was conducted using the estimated exposures in the two species without adjustment by the difference in protein binding as protein binding was the same in dog and human.

### Software

The data used in the analyses were extracted from the different referred publications using ScanIt plot digitizer software, version 2.0 [29]. The analyses were conducted using NONMEM, version 7.4 (ICON Development Solutions, Hanover, MD, USA). Graphical data presentations were conducted using R (R Foundation for Statistical Computing).

## Results

### Modeling viral load and rate of recovery in dog

Emax and Weibull models were tested as alternative models for fitting the data.

The performance of the two models was assessed using the Akaike information criterion and the inspection of the observed and model predicted data. The Weibull model was finally retained as it showed better performance in fitting either the viral load or the rate of recovery data.

**Fig. 2:**
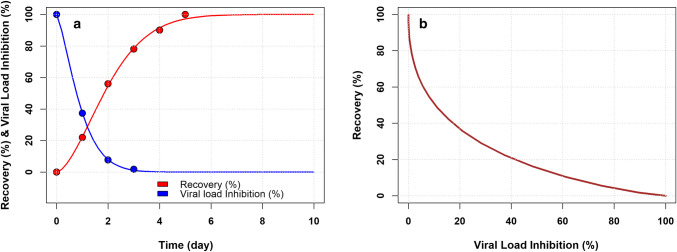
**a** Observed (dots) and model predicted (solid lines) recovery % (red) and viral load (blue) data in CCoV infected dog; **b** curvilinear relationship between viral load inhibition % and recovery % (Color figure online)

Figure 2a shows the observed and model-predicted rate of recovery [16] and viral load data [11] in CCoV infected dogs. Figure 2b shows the curvilinear relationship between viral load and rate of recovery estimated using the Weibull model predictions. The estimated parameters are presented in Table 1. The Weibull models properly described the % of recovery and viral load data. The estimated td parameters indicated that after one day of treatment INDO was able to inhibit 63% of the viral load while 63% of recovery was expected after 2.3 days of treatment.

**Table 1:** Estimated parameters (standard error) of the models describing the % of recovery and viral load data in CCoV infected dogs

Parameter	Viral load	Rate of recovery
td (day)	1.01 (< 0.001)	2.30 (< 0.001)
g (unitless)	1.36 (< 0.001)	1.64 (< 0.001)

### PK in dog and human

All the PK analyses were conducted on the mean concentration time course values of INDO in dogs and humans. The PK observations were obtained by digitizing the concentration versus time graphs reported in the referred publications.

The INDO PK in dog and human was well described by a two-compartment model with first order distribution and elimination processes. The absorption was characterized by a lag-time in human and dog, by a zero-order drug release for the IR formulation and by a first-order process for the SR formulation. The residual error was described by a combined additive and proportional error model. The estimated PK parameters in dog after an IR formulation, and in human after IR and SR formulations are presented in Table 2. The precision of all PK parameters presented in Table 2 was < 0.001. The relative high precision of the estimated parameters was probably due to the use of the mean data in the fitting procedure, to the limited number of observations with respect to the number of parameters in the model and to the limited variability on the data.

**Table 2:** Estimated parameters of the models describing the INDO PK in dog after an IR formulation, and in human after IR and SR formulations

Parameter	Dog	Human-IR	Human-SR
D1 (h)*, ka (h^−1^)**	1.36*	0.965*	0.561**
V/F (L)	3.79	11.90	15.10
kel (h^−1^)	0.77	0.51	0.05
k12 (h^−1^)	0.21	0.35	0.52
k21 (h^−1^)	0.41	0.56	0.01
Lag time (h)	0.16	0.08	0.29
Add	0.02	0.03	0^#^
Prop	0.10	0.10	0.10

The estimated standard error was < 0.001 for each parameter

*Add* residual error standard deviation of the additive error model component, *Prop* residual error standard deviation of the proportional error model component

*D1 = duration of the zero-order input, **ka absorption rate constant, ^#^fixed value

The maximal concentration was reached between 1 h and 1.5 h post-dose in dog and in human with the IR formulation and between 2 h and 2.5 h post-dose in human with the SR formulation. INDO is rapidly distributed and eliminated, the fraction of the dose (estimated as the fraction of the total AUC) cleared from the systemic circulation in the initial 6 h post-dose is 90% in dog, 84% in human with the IR formulation and 12% in human with the SR formulation. The corresponding INDO half-life in the initial 6 h was estimated ~ 3 h for the SR formulation, ~ 1.5 h for the IR formulation in human, and ~ 1 h in dog. The observed and model-predicted INDO concentrations and the 95% prediction intervals for the mean profile are presented in Supplemental Material (SFigs. 1, 2, and 3) in dog at the dose of 25 mg, in human at the dose 25 mg to 100 mg IR, and in human at the dose of 75 mg ER, respectively. The 95% prediction intervals for the mean profile were computed by simulating the model outcomes using the estimated parameters and the estimated residual error: 200 replicates of the original dataset were simulated, based on the final model, and 95% prediction intervals for the mean profile were computed based on the simulated datasets.

**Fig. 3:**
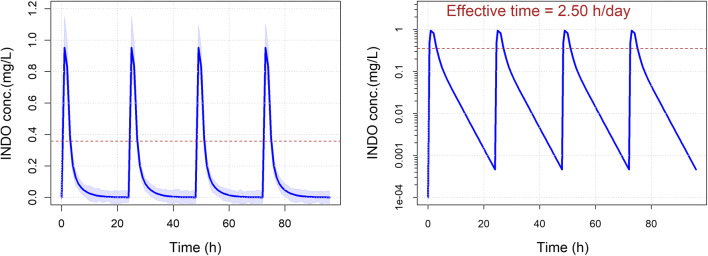
Simulated INDO exposure in dog treated with 1 mg/kg/day

No accumulation was expected in dog after repeated INDO daily administration due to the very short half-life. The simulated INDO exposure at 1 mg/kg/day (equivalent to 8.5 mg/day due to an average weight of 8.5 kg of the treated dogs) in the CCoV infected dogs after 4 days of treatment is presented in Fig. 3. This simulation indicated that identical levels of INDO with large peak-to-trough ratio were expected on each day of treatment.

### Translational model

The estimated plasma concentration in dog following an INDO daily dose of 1 mg/kg indicated a lack of accumulation due to the very short half-life of INDO. These data thus supported the hypothesis that the observed efficacy was not due to an accumulated exposure but to the time during which the exposure was above an effective value. The effective exposure, initially defined as an exposure greater or equal to the IC50 value, was estimated to 0.358 mg/L considering a molecular weight of INDO of 357.8 g/mol and an IC50 of 1 µM. The estimated time during which the exposure was above 0.358 mg/L was 2.5 h/day.

**Fig. 4:**
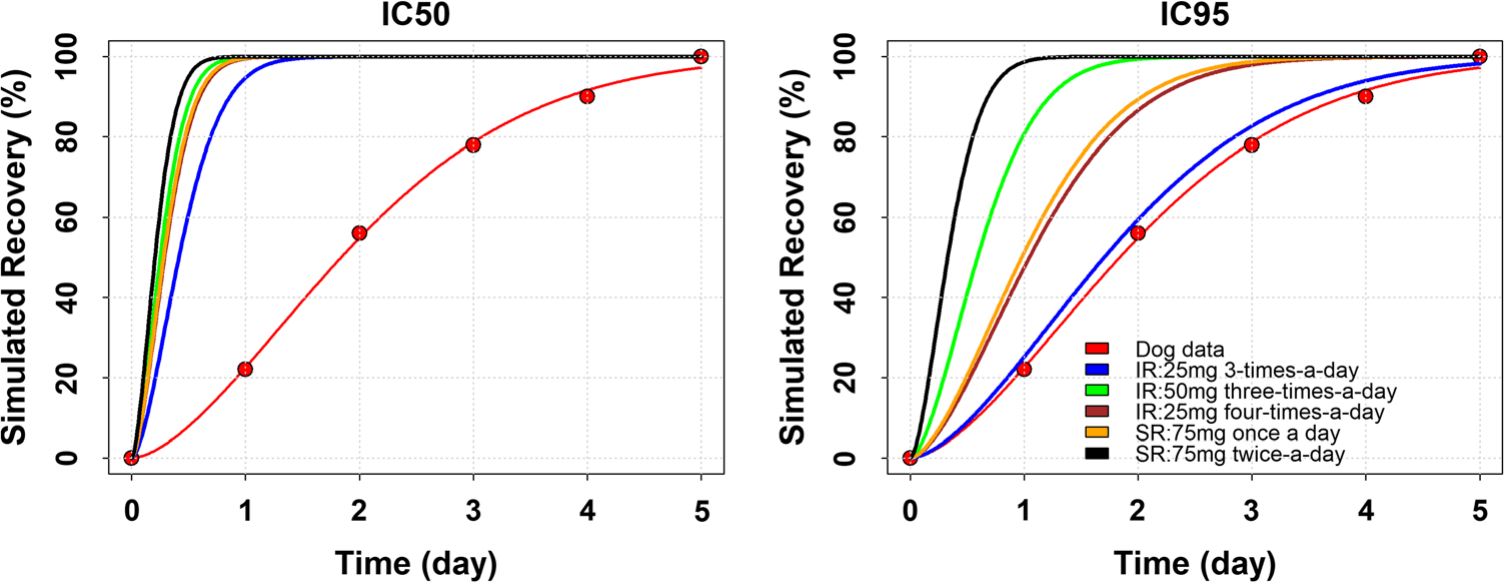
Simulated recovery % by dosage regimen as a function of the target potency (IC50 and IC95)

Two parameters were used to characterize the % of recovery: td representing the time-to-response (i.e. the time necessary for ~ 63% of the total response), and g the shape of the curve. The time during which the INDO concentration remains above the effective concentration was assumed to affect the time-to-response (the shape of the time-to-response curve was assumed invariant with respect to the INDO exposure). According to Eq. 1 two extreme scenarios can be considered to characterize the response (1) a time during which the INDO concentration remains above the effective concentration equal to zero associated with a td = 0 leading to a flat zero response and (2) a very large td value associated with an INDO concentration always above the effective concentration 24 h a day leading to a flat 100% response. Any intermediate time during which the INDO concentration remains above the effective concentration was associated with a td value providing a graded response as pictorially represented in Fig. 4.

A proportionality rule was used as a straightforward method for estimating the adjusted td in human as a function of the estimated time during which the INDO concentration was expected to remain above the effective concentration. The proportionality rule was based on the following consideration: a larger exposure was associated with a larger effective exposure, and the size of the response was proportional to the level of the effective exposure.

The expected recovery (%) in human was estimated using the recovery (%) in dog (see Table 1) with a td value adjusted for the estimated time during which the exposure in human was above the effective exposure value. The adjustment was done by assuming that larger values of td were associated with short time during which the daily exposure was above the effective exposure using a proportionality rule.

Different values of the effective exposure were estimated according to the different dosage regimens and different values of the target in-vitro potency. The INDO exposure simulated after different dosage regimens of the IR and the SR formulations during 6 days of treatment are presented in the Supplemental Material (SFigs. 4–7).

The effective exposure was estimated during 6 days of treatment as the pre-clinical data indicated that a complete response was observed in the initial 5 days of treatment.

A complete response was defined as the complete (100%) disappearance of the symptoms. The estimated times above the effective exposure by dosage regimen are presented in Table 3 as a function of the target potency associated with the IC50 and IC95 values. The effective exposure for the IR formulation was the same on each day of treatment due to the lack of accumulation differently from the values of the SR formulation. The repeated administration of the SR formulation was associated with increased exposure leading to an increase of the effective exposure value with time.

**Table 3:** Time/day above the effective exposure by dosage regimen as a function of the target potency (IC50 and IC95)

	Dose (mg)	Dosage regimen	Time above effective exposure (h/day)	
			IC50	IC95
IR formulation	25	Three-times-a-day	11.10	2.70
	50	Three-times-a-day	18.30	7.80
	25	Four-times-a-day	16.00	4.40
SR formulation	75	Once-a-day	16.47	4.70
	75	Twice-a-day	22.50	14.03

The adjusted td values by dosage regimen are presented in Table 4 as a function of the target potency associated with the IC50 and IC95 values. A lower td value was associated with an earlier onset of response. Therefore, the best performing dosage was 75 mg SR formulation BID and the worst dosage was 25 mg IR formulation TID.

**Table 4:** Estimated td value by dosage regimen and formulation as a function of target potency

	Dose (mg)	Dosage regimen	Adjusted td (h)	
			IC50	IC95
IR formulation	25	Three-times-a-day	0.52	2.13
	50	Three-times-a-day	0.31	0.74
	25	Four-times-a-day	0.36	1.31
SR formulation	75	Once-a-day	0.35	1.22
	75	Twice-a-day	0.26	0.41

The expected % of recovery in human by dosage regimen are presented in Fig. 4 as a function of the target potency associated with the IC50 and IC95 values. The simulated % of recovery in human was always faster than the one observed in animal. This because the time above the effective exposure in human (= 2.7 h/day in the worst-case scenario for 25 mg IR TID) was always larger than the time above the effective exposure in dog (= 2.5 h/day).

## Discussion

The aim of this study was not to prove the efficacy of INDO for the treatment of SARS-CoV-2 but to show that INDO could be considered as a promising candidate for the treatment of patients infected with the SARS-COV-2 virus based on the evidence derived from studies conducted in-vitro, in animals, and on the model-based simulations.

The Food and Drug administration (FDA) approved INDO in 1965 under the brand name Indocin^®^. INDO is approved to treat moderate to severe osteoarthritis, rheumatoid arthritis, and ankylosing spondylitis. Today, several drug manufacturers make generic versions of the drug. INDO was also found to have significant anticancer activity against a wide variety of cancer cell types, in vitro and in vivo [30, 31]. INDO performs its anticancer activity in different fashions, inhibits proliferation via induction of apoptotic death of tumor cells [29, 31], reduces tumorigenesis by enhancing the immune response [32, 33] and inhibiting the angiogenesis [34] as well. Recently, extensive studies on various cancer cell types including colorectal carcinoma justified the efficacy of INDO to reduce the levels of anti-apoptotic proteins and progressive cell proliferation [35, 36].

Coronavirus is an envelope virus with four structural proteins: spike (S) protein, membrane (M) protein, envelope (E) protein, and nucleocapsid (N) protein [37]. S protein is responsible for the virus attachment and entry to the target cells, which initiate the infection process. S protein plays key roles in the induction of protective humoral and cellular immunity during SARS-CoV. This is the reason why the pseudovirus model that contains the SARS-CoV-2 spike was considered as the most attractive target for SARS-CoV vaccine and therapeutic development [38, 39].

In-vitro, INDO was found to be active against several viruses, including SARS-CoV-1 virus, and SARS-CoV-2 pseudovirus [11, 16]. This antiviral activity was also shown in CCoV and in several other RNA-viruses suggesting a cellular rather than a viral target for the drug [11, 16].

The in-vitro results presented in this paper indicated that INDO is active in the human SARS-CoV-2 pseudovirus at low micromolar range, that a good correlation exists between viral load and rate of response in CCoV infected dogs, that INDO has a similar in-vitro inhibitory effect on SARS-CoV-2 pseudovirus and CCov, and that a good expectation exists for the human performance of INDO in the treatment of patients infected with SARS-CoV-2.

A meta-analysis recently conducted on 9 studies including laboratory-confirmed 1426 patients affected by SARS-CoV-2 suggests that there were mild or severe cytokine storm in severe patients, which was an important cause of death. Therefore, the treatment of cytokine storm has become an important part of rescuing severe patients. Interleukin-6 (IL-6) plays an important role in cytokine release syndrome. If a drug can block the signal transduction pathway of IL-6, it is expected to become a new treatment of severe patients [40].

To explore the role of IL-6 in the SARS-CoV-2 infection, the CORIMUNO-TOCI trial was conducted using tocilizumab (clinicaltrials.gov reference NCT04331808). Tocilizumab is a blocker of IL-6R, which can effectively block IL-6 signal transduction pathway [41, 42]. The results of this study have yet to be published, but the outcomes were reported in a press release. It included patients who were hospitalized with SARS-COV-2 moderate to severe pneumonia in intensive care or at high risk of requiring intensive care but did not need resuscitation upon admission. The study included 129 patients who were randomized to either usual treatment plus tocilizumab (n = 65) or usual treatment alone (n = 64).

One of the interesting pharmacological effects of INDO is its modulation of cytokine production [43] and its robustly effect on the reduction of proinflammatory IL-6 as shown in a study conducted in mice where a decrease of 75% to 80% of IL-6 has been observed [44]. This feature in combination with the antiviral properties of INDO on human SARS-CoV-1, canine CCoV, and SARS-CoV-2 [11, 16], further highlight how INDO could be used to potentially aid the fight against the coronavirus.

Different simulations were conducted to evaluate the expected performances of different dosage regimens using the time during which the INDO exposure remains above the effective concentration as a criterion for assessing efficacy.

Two thresholds for the effective INDO concentration were considered: the first one (best case scenario) was associated with the concentration at which pseudovirus replication is inhibited by 50% (i.e. 0.358 mg/L) and the second one (worst case scenario) associated with the concentration at which virus replication is inhibited by 95% (i.e. 1.074 mg/L).

The results of the analysis indicated that the 75 mg SR BID dosage regimen was expected to deliver an improved clinical benefit given the larger time/day during which the exposure was expected to remain above the effective concentration following this dosage regimen.

The doses used in the simulations were selected based on the recommended dosage regimen of INDO reported in the label for the IR [9] and the SR formulations [10]. The recommended dosage for the IR formulation is 25 mg BID or TID. If this is well tolerated, the daily dosage can be increased by 25 mg or by 50 mg, but the total daily dose should not exceed 200 mg. INDO SR, 75 mg once a day can be substituted for INDO IR capsules, 25 mg three times a day; and INDO SR, 75 mg BID can be substituted for INDO IR 50 mg three times a day.

Among the limitations that could affect the assessment of the INDO effect:

1. The in-vitro test showed that INDO had a directly and potently antiviral activity against the SARS CoV-2 pseudovirus and not on the SARS Cov-2 virus. The pseudovirus model that contains the SARS-CoV-2 spike was used to study the S/receptor interactions and this test is not necessarily indicative of an effect on virus replication;
2. The assumption that the broad range effects on coronaviruses (e.g., SARS-CoV-1, CCoV, and SARS-CoV-2) can be used to predict the INDO efficacy in the treatment of SARS-CoV-2;
3. The assumption that INDO inhibits viral replication of human SARS-CoV-2 at the same concentration that inhibits CCoV;
4. There are no deep pools of the virus and what is in the blood reflects what is in the target;
5. The assumption that the time above the effective concentration is the driver of the efficacy;
6. The limited number of dogs used to assess viral load and efficacy in animal studies;
7. The use of the proportionality criterion in the translational model for estimating the clinical response in human.

The limitations of the present analyses mainly concern the reliability and the predictive performances of the outcomes of the animal and in-vitro studies. The relevance of these studies mainly concerns the proof of mechanism of new candidate drugs. These studies provide only indicative information on the potential performances of new treatments in controlled clinical trials as shown by the outcomes of recent clinical trials on HCQ and remdesivir [45–47]. Two drugs with an in-vitro potency very closed to the in-vitro potency of INDO [17, 18].

## Conclusions

The pharmacological properties of INDO suggest that INDO could exert both anti-inflammatory (reduction of proinflammatory IL-6) and antiviral effects when used for treating patients infected with SARS-CoV-2. The analyses presented in this paper suggest that INDO treatment with the SR formulation at the dose of 75 mg twice-a-day is expected to achieve a complete response in three days for the treatment of patients infected by the SARS-CoV-2 coronavirus. These results suggest that INDO could be considered as a promising candidate for the treatment of SARS-CoV-2 whose potential therapeutic effect needs to be further assessed in a prospective clinical trial.

As very recent studies conducted on remdesivir and HCQ (two among the most promising treatments) failed to demonstrate efficacy in patients hospitalized for a documented SARS-CoV-2 pneumonia [45–47]; the findings of the present analysis could be considered as particularly critical for defining new approaches for the battle against this major endemic disease.

## Electronic supplementary material

Below is the link to the electronic supplementary material.

Supplementary file1 (PDF 229 kb)
