# Supplementary material for: Model based approach for estimating the dosage regimen of indomethacin a potential antiviral treatment of patients infected with SARS CoV-2
Source: J Pharmacokinet Pharmacodyn. 2020 May 20;47(3):189–98. doi: 10.1007/s10928-020-09690-4 (PMC7237801; doi:10.1007/s10928-020-09690-4)
Supplement: Supplementary file 1 — Supplementary file1 (PDF 229 kb) [file 10928_2020_9690_MOESM1_ESM.pdf]

Model Based Approach for Estimating the Dosage Regimen of Indomethacin a Potential Antiviral Treatment of Patients Infected with SARS CoV-2

<u>Supplementary Material</u>

SFig 1. Observed (dots) and model predicted (solid line) INDO concentrations in dog at the dose of 25 mg. The shaded area represents the 95% prediction interval.

SFig 2. Observed (dots) and model predicted (solid line) dose normalized INDO IR concentrations following the doses of 25 mg, 50 mg, 75 mg, and 100 mg. The shaded area represents the 95% prediction interval.

SFig 3. Observed (dots) and model predicted (solid line) INDO concentrations at the dose of 75 mg of the SR formulation. The shaded area represents the 95% prediction interval.

SFig 4. Target potency IC50 – IR formulation - Simulated INDO exposure after different dosage regimens. The horizontal dotted line corresponds to the target exposure level.

SFig 5. Target potency IC50 – SR formulation - Simulated INDO exposure after different dosage regimens. The horizontal dotted line corresponds to the target exposure level.

SFig 6. Target potency IC95 – IR formulation - Simulated INDO exposure after different dosage regimens. The horizontal dotted line corresponds to the target exposure level.

SFig 7. Target potency IC95 – SR formulation - Simulated INDO exposure after different dosage regimens. The horizontal dotted line corresponds to the target exposure level.

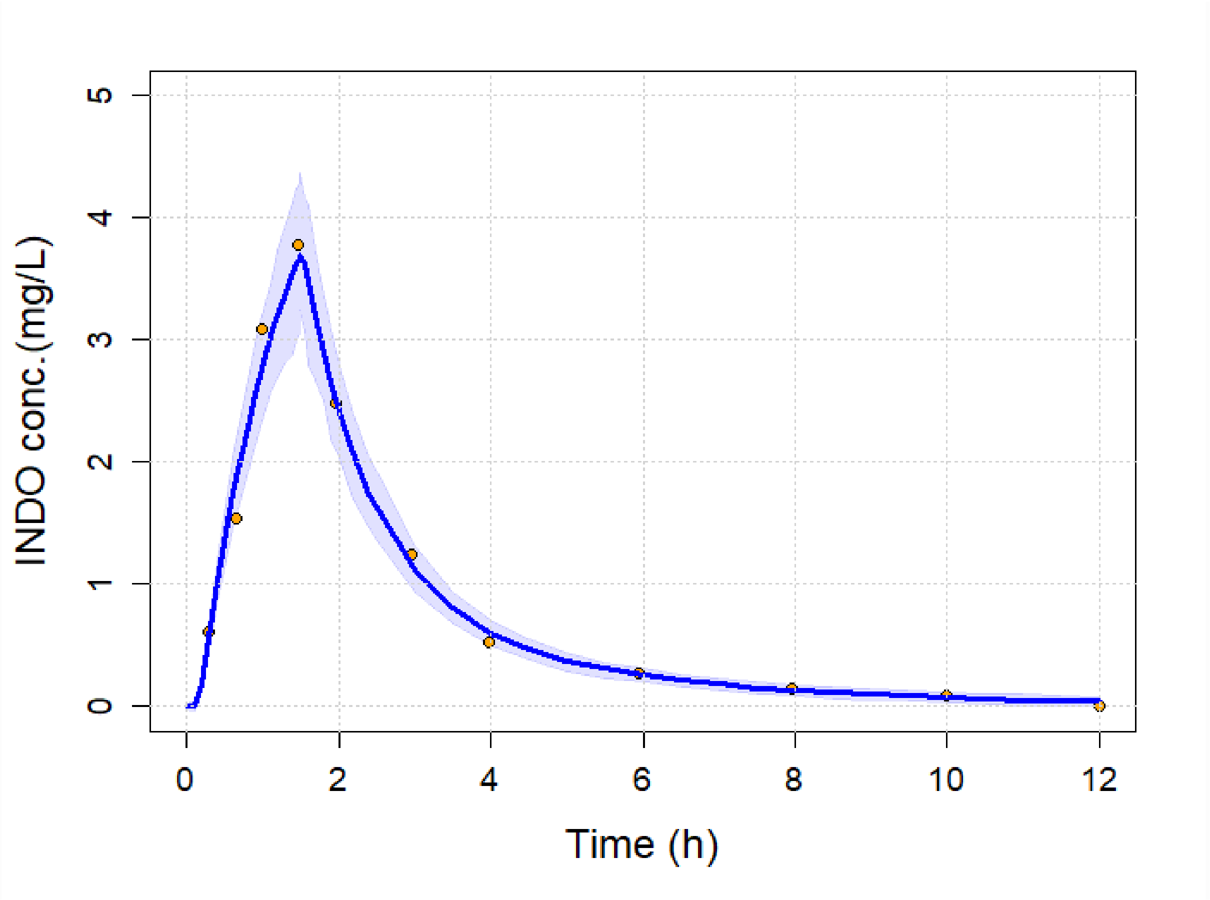

SFig 1. Observed (dots) and model predicted (solid line) INDO concentrations in dog at the dose of 25 mg. The shaded area represents the 95% prediction interval.

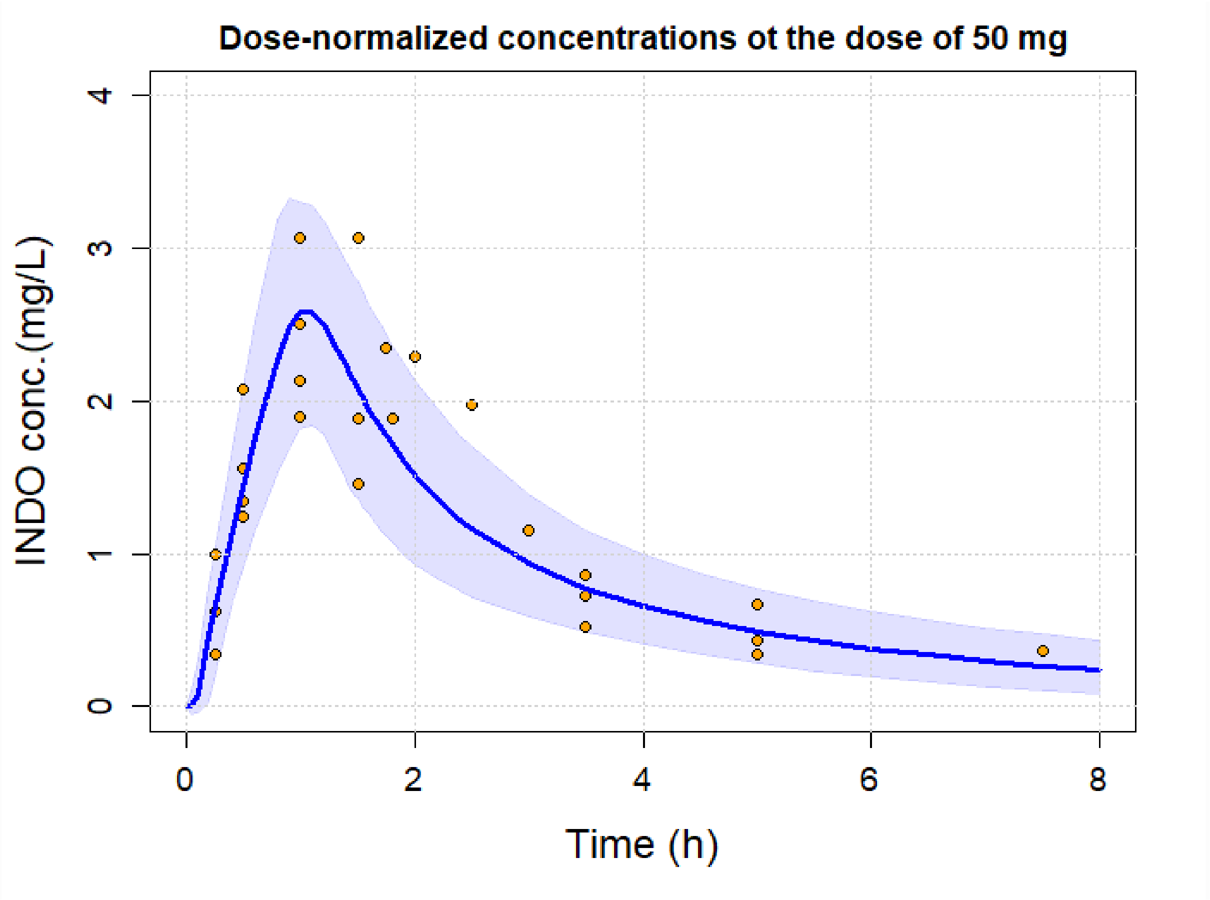

SFig 2. Observed (dots) and model predicted (solid line) dose normalized INDO IR concentrations following the doses of 25 mg, 50 mg, 75 mg, and 100 mg. The shaded area represents the 95% prediction interval

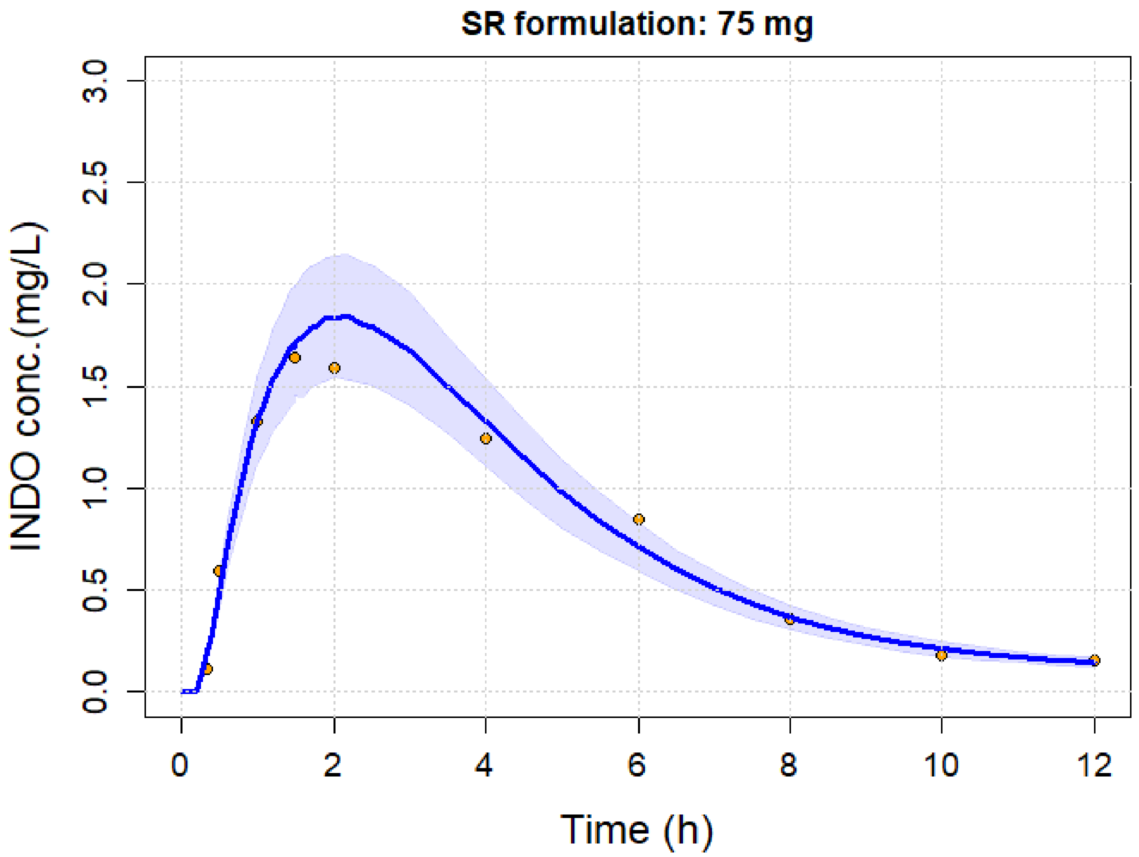

SFig 3. Observed (dots) and model predicted (solid line) INDO concentrations at the dose of 75 mg of the SR formulation. The shaded area represents the 95% prediction interval.

.

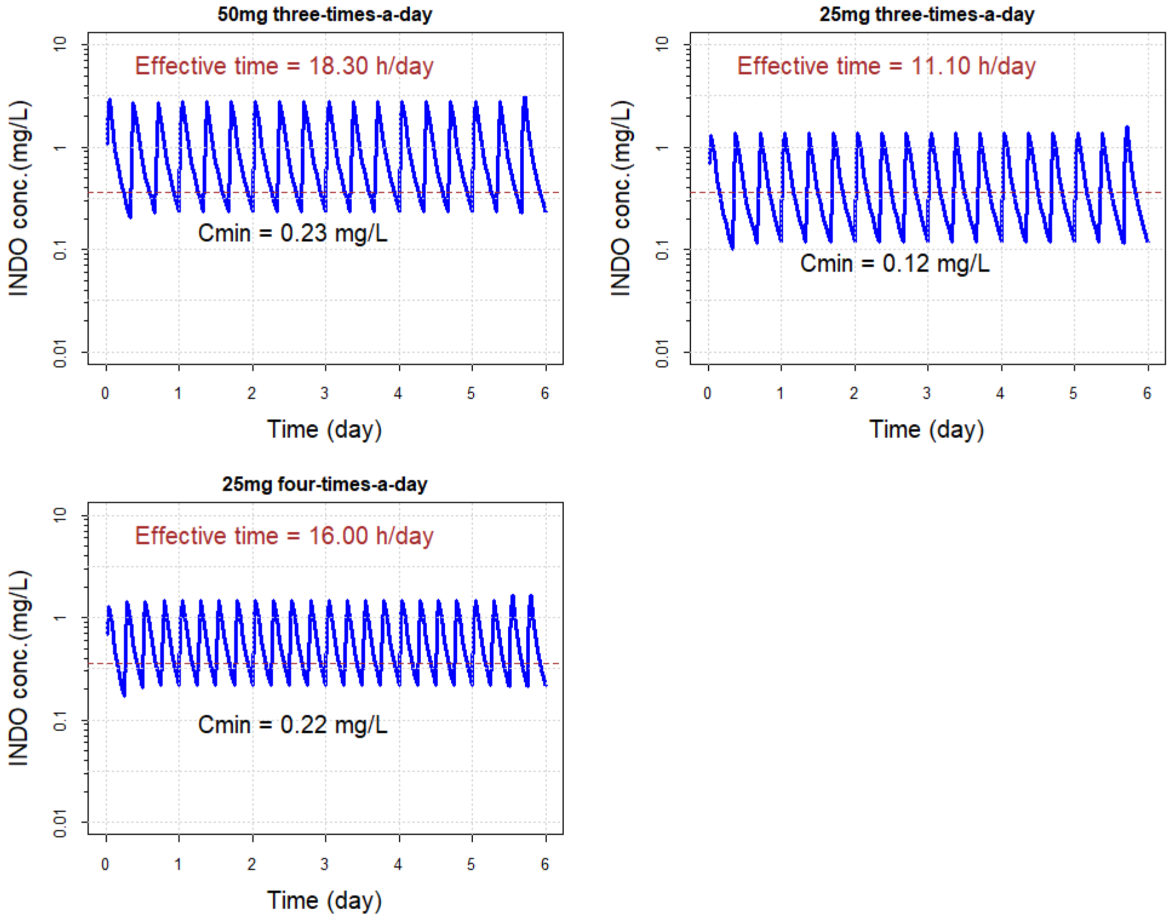

SFig 4. Target potency IC50 – IR formulation - Simulated INDO exposure after different dosage regimens. The horizontal dotted line corresponds to the target exposure level.

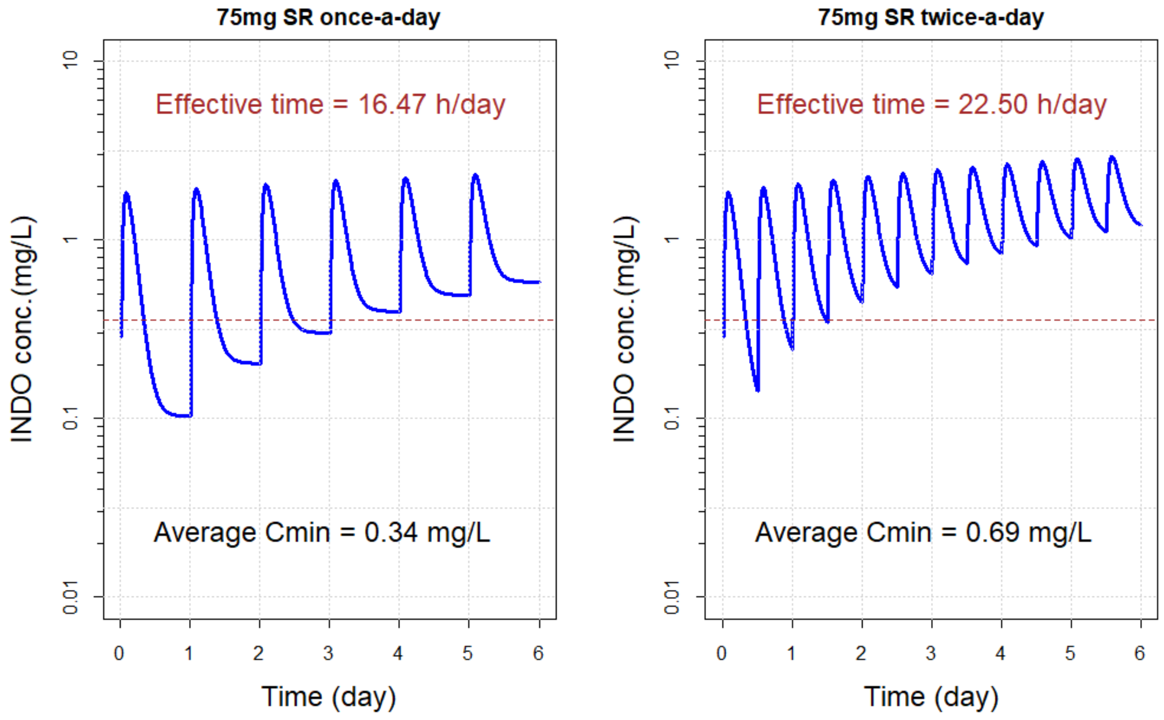

SFig 5. Target potency IC50 – SR formulation - Simulated INDO exposure after different dosage regimens. The horizontal dotted line corresponds to the target exposure level.

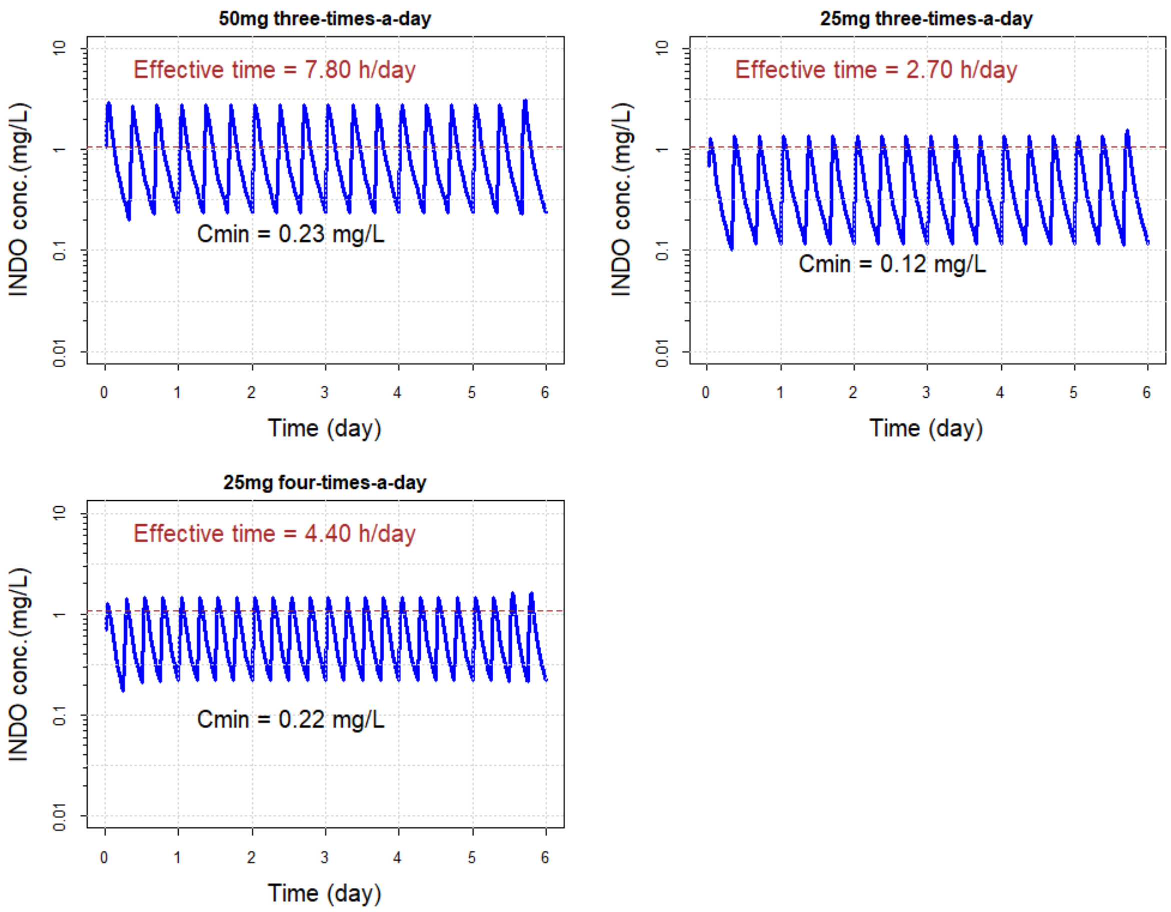

SFig 6. Target potency IC95 – IR formulation - Simulated INDO exposure after different dosage regimens. The horizontal dotted line corresponds to the target exposure level.

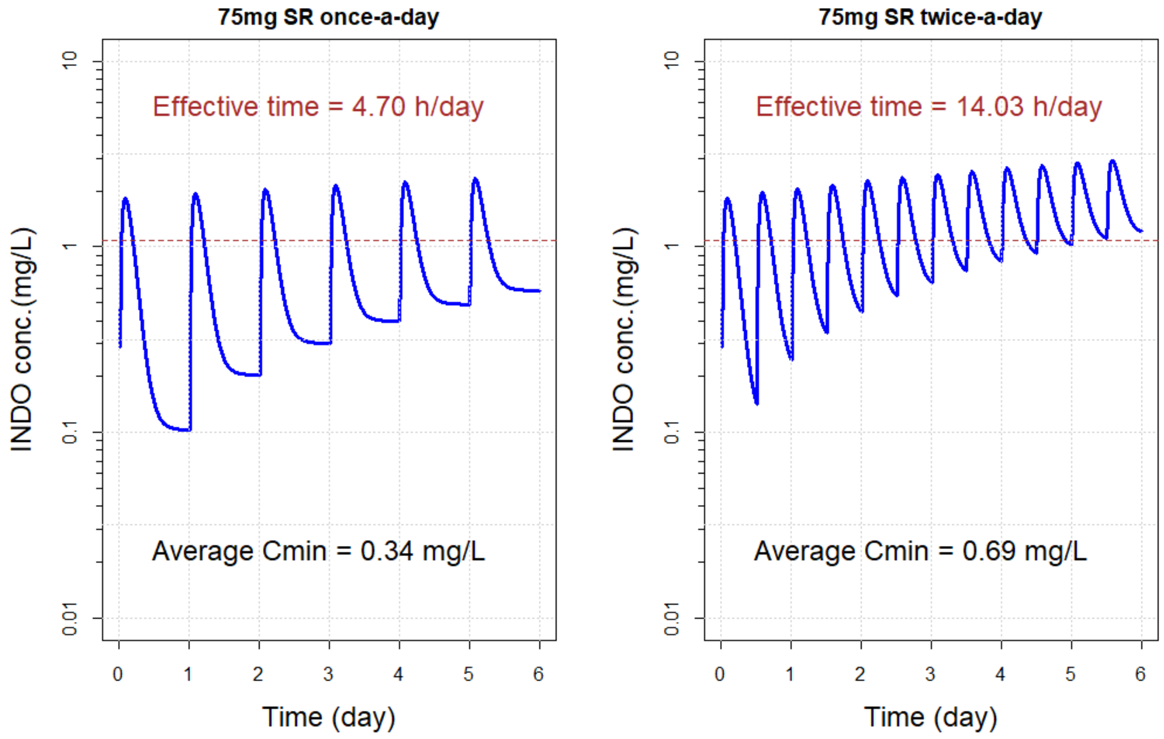

SFig 7. Target potency IC95 – SR formulation - Simulated INDO exposure after different dosage regimens. The horizontal dotted line corresponds to the target exposure level.
